# 3-(5-Methyl-2-fur­yl)-1-(*p*-tol­yl)-2-propen-1-one

**DOI:** 10.1107/S1600536808029152

**Published:** 2008-09-17

**Authors:** Huan-Mei Guo, Xian-Bing Wang, Fang-Fang Jian

**Affiliations:** a Microscale Science Institute, Department of Chemistry and Chemical Engineering, Weifang University, Weifang 261061, People’s Republic of China; b Department of Equipment, Weifang University, Weifang 261061, People’s Republic of China; c Microscale Science Institute, Weifang University, Weifang 261061, People’s Republic of China

## Abstract

The title compound, C_15_H_14_O_2_, was prepared from 4-methyl­hypnone and 5-methyl­furfural by Clasion–Schmidt condensation. All of the bond lengths and bond angles are in normal ranges. The dihedral angle formed by the benzene ring and furan ring is 5.31 (2).

## Related literature

For the biological activity of chalcones, see: Hsieh *et al.* (1998 ▶); Anto *et al.* (1994 ▶). For the effectiveness of chalcones against cancer, see: De Vincenzo *et al.* (2000 ▶); Dimmock *et al.* (1998 ▶). For bond-length and angle data, see: Ali *et al.* (2005 ▶); Zhou (2007 ▶).
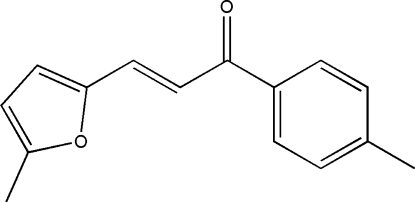


## Experimental

### 

#### Crystal data

C_15_H_14_O_2_
*M* *_r_* = 226.26
Monoclinic, 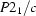

*a* = 8.0394 (8) Å
*b* = 17.0278 (17) Å
*c* = 10.6550 (8) Å
β = 121.347 (6)°
*V* = 1245.7 (2) Å^3^
*Z* = 4
Mo *K*α radiation
μ = 0.08 mm^−1^
*T* = 293 (2) K
0.2 × 0.2 × 0.2 mm

#### Data collection

Bruker SMART CCD area-detector diffractometer
Absorption correction: none
7980 measured reflections
2985 independent reflections
1706 reflections with *I* > 2σ(*I*)
*R*_int_ = 0.026

#### Refinement

*R*[*F* ^2^ > 2σ(*F* ^2^)] = 0.043
*wR*(*F* ^2^) = 0.136
*S* = 1.02
2985 reflections
155 parameters
H-atom parameters constrained
Δρ_max_ = 0.17 e Å^−3^
Δρ_min_ = −0.11 e Å^−3^

### 

Data collection: *SMART* (Bruker, 1997 ▶); cell refinement: *SAINT* (Bruker, 1997 ▶); data reduction: *SAINT*; program(s) used to solve structure: *SHELXS97* (Sheldrick, 2008 ▶); program(s) used to refine structure: *SHELXL97* (Sheldrick, 2008 ▶); molecular graphics: *SHELXTL* (Sheldrick, 2008 ▶); software used to prepare material for publication: *SHELXTL*.

## Supplementary Material

Crystal structure: contains datablocks I. DOI: 10.1107/S1600536808029152/at2615sup1.cif

Structure factors: contains datablocks I. DOI: 10.1107/S1600536808029152/at2615Isup2.hkl

Additional supplementary materials:  crystallographic information; 3D view; checkCIF report

## Figures and Tables

**Fig. 1.**
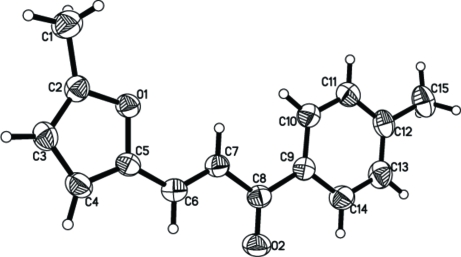
The structure of the title compound showing 30% probability displacement ellipsoids and the atom-numbering scheme.

## supplementary crystallographic information

### Comment

Among flavonoids, chalcones have been identified as interesting compounds having multiple biological actions which include antiinflammatory (Hsieh *et al.*,1998) and antioxidant (Anto *et al.*, 1994). Of particular interest, the effectiveness of chalcones against cancer has been investigated (De Vincenzo *et al.*, 2000; Dimmock *et al.*, 1998). As part of our search for new biologically active compounds we synthesized the title compound (I), and describe its structure here.

In the structure of (I) (Fig. 1), all of the bond lengths and bond angles fall in the normal range (Zhou, 2007; Ali *et al.*, 2005). The dihedral angles formed by the benzene ring and furan ring is 5.31 (2)°. There are some weak C—H···O hydrogen bonds in the crystal structure (Table 1).

### Experimental

A mixture of the 5-methylfurfural (0.02 mol), and 4-methylhypnone (0.02 mol) and 10% NaOH (10 ml) was stirred in ethanol (30 mL) for 3 h to afford the title compound (yield 85%). Single crystals suitable for X-ray measurements were obtained by recrystallization from ethanol at room temperature.

### Refinement

H atoms were fixed geometrically and allowed to ride on their attached atoms, with C—H distances = 0.93-0.96 Å, and with *U*_iso_=1.2–1.5*U*_eq_.

### Crystal data

**Table:**

C_15_H_14_O_2_	*F*(000) = 480
*M**_r_* = 226.26	*D*_x_ = 1.206 Mg m^−^^3^
Monoclinic, *P*2_1_/*c*	Mo *K*α radiation, λ = 0.71073 Å
Hall symbol: -P 2ybc	Cell parameters from 1770 reflections
*a* = 8.0394 (8) Å	θ = 0.4–27.5°
*b* = 17.0278 (17) Å	µ = 0.08 mm^−^^1^
*c* = 10.6550 (8) Å	*T* = 293 K
β = 121.347 (6)°	Bar, colourless
*V* = 1245.7 (2) Å^3^	0.2 × 0.2 × 0.2 mm
*Z* = 4	

### Data collection

**Table:**

Bruker SMART CCD area-detector diffractometer	1706 reflections with *I* > 2σ(*I*)
Radiation source: fine-focus sealed tube	*R*_int_ = 0.026
graphite	θ_max_ = 28.2°, θ_min_ = 2.4°
phi and ω scans	*h* = −8→10
7980 measured reflections	*k* = −17→22
2985 independent reflections	*l* = −13→13

### Refinement

**Table:**

Refinement on *F*^2^	Secondary atom site location: difference Fourier map
Least-squares matrix: full	Hydrogen site location: inferred from neighbouring sites
*R*[*F*^2^ > 2σ(*F*^2^)] = 0.043	H-atom parameters constrained
*wR*(*F*^2^) = 0.136	*w* = 1/[σ^2^(*F*_o_^2^) + (0.0613*P*)^2^ + 0.0738*P*] where *P* = (*F*_o_^2^ + 2*F*_c_^2^)/3
*S* = 1.02	(Δ/σ)_max_ < 0.001
2985 reflections	Δρ_max_ = 0.17 e Å^−^^3^
155 parameters	Δρ_min_ = −0.11 e Å^−^^3^
0 restraints	Extinction correction: *SHELXL*, Fc^*^=kFc[1+0.001xFc^2^λ^3^/sin(2θ)]^-1/4^
Primary atom site location: structure-invariant direct methods	Extinction coefficient: 0.011 (3)

### Special details

**Table:**

Geometry. All e.s.d.'s (except the e.s.d. in the dihedral angle between two l.s. planes) are estimated using the full covariance matrix. The cell e.s.d.'s are taken into account individually in the estimation of e.s.d.'s in distances, angles and torsion angles; correlations between e.s.d.'s in cell parameters are only used when they are defined by crystal symmetry. An approximate (isotropic) treatment of cell e.s.d.'s is used for estimating e.s.d.'s involving l.s. planes.
Refinement. Refinement of *F*^2^ against ALL reflections. The weighted *R*-factor *wR* and goodness of fit *S* are based on *F*^2^, conventional *R*-factors *R* are based on *F*, with *F* set to zero for negative *F*^2^. The threshold expression of *F*^2^ > σ(*F*^2^) is used only for calculating *R*-factors(gt) *etc*. and is not relevant to the choice of reflections for refinement. *R*-factors based on *F*^2^ are statistically about twice as large as those based on *F*, and *R*- factors based on ALL data will be even larger.

### Fractional atomic coordinates and isotropic or equivalent isotropic displacement parameters (Å^2^)

**Table:**

	*x*	*y*	*z*	*U*_iso_*/*U*_eq_	
O1	−0.32694 (14)	−0.22464 (6)	−0.56944 (10)	0.0574 (3)	
O2	0.14482 (19)	−0.01503 (7)	−0.24561 (13)	0.0810 (4)	
C1	−0.5507 (3)	−0.33021 (11)	−0.7020 (2)	0.0892 (6)	
H1A	−0.6226	−0.3521	−0.7991	0.134*	
H1B	−0.6395	−0.3140	−0.6722	0.134*	
H1C	−0.4632	−0.3691	−0.6347	0.134*	
C2	−0.4379 (2)	−0.26168 (9)	−0.70182 (17)	0.0582 (4)	
C3	−0.4181 (2)	−0.22529 (10)	−0.80412 (18)	0.0656 (4)	
H3A	−0.4779	−0.2393	−0.9026	0.079*	
C4	−0.2899 (3)	−0.16169 (10)	−0.73553 (18)	0.0665 (4)	
H4A	−0.2497	−0.1259	−0.7804	0.080*	
C5	−0.2364 (2)	−0.16241 (8)	−0.59293 (16)	0.0545 (4)	
C6	−0.1121 (2)	−0.11417 (9)	−0.47132 (18)	0.0590 (4)	
H6A	−0.0541	−0.0723	−0.4903	0.071*	
C7	−0.0686 (2)	−0.12165 (9)	−0.33318 (17)	0.0577 (4)	
H7A	−0.1275	−0.1613	−0.3099	0.069*	
C8	0.0699 (2)	−0.06898 (9)	−0.21687 (17)	0.0591 (4)	
C9	0.1224 (2)	−0.08231 (9)	−0.06208 (16)	0.0558 (4)	
C10	0.0379 (2)	−0.13889 (10)	−0.02027 (18)	0.0683 (5)	
H10A	−0.0550	−0.1724	−0.0908	0.082*	
C11	0.0888 (3)	−0.14648 (11)	0.1236 (2)	0.0761 (5)	
H11A	0.0287	−0.1850	0.1483	0.091*	
C12	0.2260 (3)	−0.09881 (10)	0.23223 (19)	0.0701 (5)	
C13	0.3126 (3)	−0.04334 (11)	0.1911 (2)	0.0802 (5)	
H13A	0.4077	−0.0108	0.2624	0.096*	
C14	0.2624 (3)	−0.03474 (10)	0.04727 (19)	0.0734 (5)	
H14A	0.3235	0.0036	0.0231	0.088*	
C15	0.2803 (3)	−0.10657 (13)	0.3896 (2)	0.0940 (6)	
H15A	0.2064	−0.1483	0.3979	0.141*	
H15B	0.2525	−0.0582	0.4215	0.141*	
H15C	0.4168	−0.1181	0.4500	0.141*	

### Atomic displacement parameters (Å^2^)

**Table:**

	*U*^11^	*U*^22^	*U*^33^	*U*^12^	*U*^13^	*U*^23^
O1	0.0672 (6)	0.0599 (6)	0.0517 (6)	−0.0065 (5)	0.0355 (5)	−0.0031 (5)
O2	0.1026 (9)	0.0684 (7)	0.0750 (8)	−0.0265 (7)	0.0484 (7)	−0.0067 (6)
C1	0.1063 (15)	0.0854 (13)	0.0788 (13)	−0.0308 (11)	0.0503 (11)	−0.0160 (10)
C2	0.0607 (9)	0.0627 (9)	0.0534 (9)	−0.0021 (7)	0.0313 (7)	−0.0068 (7)
C3	0.0744 (10)	0.0738 (10)	0.0502 (9)	0.0001 (9)	0.0335 (8)	−0.0025 (8)
C4	0.0812 (11)	0.0691 (10)	0.0588 (10)	−0.0043 (9)	0.0430 (9)	0.0049 (8)
C5	0.0587 (9)	0.0546 (9)	0.0566 (9)	0.0013 (7)	0.0345 (7)	0.0025 (7)
C6	0.0630 (9)	0.0544 (9)	0.0658 (10)	−0.0025 (7)	0.0379 (8)	−0.0015 (7)
C7	0.0614 (9)	0.0544 (9)	0.0602 (10)	−0.0044 (7)	0.0337 (8)	−0.0019 (7)
C8	0.0624 (9)	0.0513 (9)	0.0649 (10)	−0.0011 (7)	0.0341 (8)	−0.0021 (7)
C9	0.0567 (9)	0.0517 (8)	0.0568 (9)	0.0029 (7)	0.0280 (7)	−0.0030 (7)
C10	0.0672 (10)	0.0725 (11)	0.0608 (11)	−0.0082 (8)	0.0302 (8)	−0.0020 (8)
C11	0.0758 (12)	0.0849 (12)	0.0676 (12)	−0.0017 (10)	0.0374 (10)	0.0088 (9)
C12	0.0721 (11)	0.0747 (11)	0.0597 (11)	0.0223 (9)	0.0315 (9)	0.0060 (8)
C13	0.0872 (13)	0.0762 (12)	0.0610 (11)	−0.0049 (10)	0.0273 (10)	−0.0138 (9)
C14	0.0856 (12)	0.0631 (10)	0.0683 (12)	−0.0131 (9)	0.0378 (10)	−0.0096 (8)
C15	0.1016 (14)	0.1128 (16)	0.0632 (12)	0.0270 (12)	0.0398 (11)	0.0108 (10)

### Geometric parameters (Å, °)

**Table:**

O1—C2	1.3689 (17)	C7—H7A	0.9300
O1—C5	1.3800 (16)	C8—C9	1.492 (2)
O2—C8	1.2218 (17)	C9—C10	1.379 (2)
C1—C2	1.477 (2)	C9—C14	1.384 (2)
C1—H1A	0.9600	C10—C11	1.372 (2)
C1—H1B	0.9600	C10—H10A	0.9300
C1—H1C	0.9600	C11—C12	1.374 (2)
C2—C3	1.333 (2)	C11—H11A	0.9300
C3—C4	1.409 (2)	C12—C13	1.374 (2)
C3—H3A	0.9300	C12—C15	1.503 (2)
C4—C5	1.347 (2)	C13—C14	1.374 (2)
C4—H4A	0.9300	C13—H13A	0.9300
C5—C6	1.416 (2)	C14—H14A	0.9300
C6—C7	1.330 (2)	C15—H15A	0.9600
C6—H6A	0.9300	C15—H15B	0.9600
C7—C8	1.464 (2)	C15—H15C	0.9600
			
C2—O1—C5	106.82 (11)	O2—C8—C9	119.91 (14)
C2—C1—H1A	109.5	C7—C8—C9	119.74 (14)
C2—C1—H1B	109.5	C10—C9—C14	117.28 (15)
H1A—C1—H1B	109.5	C10—C9—C8	124.02 (14)
C2—C1—H1C	109.5	C14—C9—C8	118.69 (15)
H1A—C1—H1C	109.5	C11—C10—C9	121.08 (16)
H1B—C1—H1C	109.5	C11—C10—H10A	119.5
C3—C2—O1	109.70 (14)	C9—C10—H10A	119.5
C3—C2—C1	134.46 (16)	C10—C11—C12	121.87 (17)
O1—C2—C1	115.83 (13)	C10—C11—H11A	119.1
C2—C3—C4	107.42 (15)	C12—C11—H11A	119.1
C2—C3—H3A	126.3	C11—C12—C13	117.08 (17)
C4—C3—H3A	126.3	C11—C12—C15	121.90 (18)
C5—C4—C3	107.23 (14)	C13—C12—C15	121.02 (18)
C5—C4—H4A	126.4	C12—C13—C14	121.72 (17)
C3—C4—H4A	126.4	C12—C13—H13A	119.1
C4—C5—O1	108.82 (13)	C14—C13—H13A	119.1
C4—C5—C6	133.33 (14)	C13—C14—C9	120.96 (17)
O1—C5—C6	117.85 (13)	C13—C14—H14A	119.5
C7—C6—C5	127.68 (15)	C9—C14—H14A	119.5
C7—C6—H6A	116.2	C12—C15—H15A	109.5
C5—C6—H6A	116.2	C12—C15—H15B	109.5
C6—C7—C8	121.64 (14)	H15A—C15—H15B	109.5
C6—C7—H7A	119.2	C12—C15—H15C	109.5
C8—C7—H7A	119.2	H15A—C15—H15C	109.5
O2—C8—C7	120.35 (15)	H15B—C15—H15C	109.5
